# Proceedings of the Thirteenth Annual Meeting of the American Dental Association

**Published:** 1873-10

**Authors:** 


AMERICAN JOURNAL

OF

DENTAL SCIENCE.

Vol. VII. THIRD SERIES--OCTOBER, 1873. No. 6.

### ARTICLE I.

### *American Dental Association.*

The thirteenth annual session of the American Dental Association was held at Put-in-Bay, commencing August 5th, 1873. The Association was called to order by the President, Dr. P. G. C. Hunt, at 10 A. M. The following named Associations, Societies and Colleges were represented:

California State Dental Association, Pennsylvania Association of Dental Surgery, Southern Dental Association, Ohio State Dental Association, Kentucky State Dental Association, Indiana State Dental Association, Missouri State Dental Association, Michigan State Dental Association, New Orleans Dental Association, Susquehannah Dental Association, Mississippi Valley Dental Association, Chicago State Dental Society, Illinois State Dental Society, St, Louis Dental Society, New York Dental Society, (6th District), Pennsylvania State Dental Society, Harvard Dental School, Philadelphia Dental College, Missouri Dental College, New York Dental College, Pennsylvania Dental College, Ohio Dental College.

Some time was consumed in receiving and examining credentials, paying dues, &c., after which the regular order of business was taken up. Dr. M. S. Dean read a report on Physiology.

After a few prefatory and apologetic lines, the writer of this report noticed briefly some new views advanced by C. S. Tomes, of London, "On the Developmental Origin of the V-shaped Maxilla," published in the *Monthly Review*. A condensed synopsis, only, was given, and the perusal of the article recommended.

The writer next briefly reviewed two articles written by H. S. Chase, which appeared in this journal—in the May and June numbers, 1873— one of which was entitled "Physiology," and the other "The part which Vital Action Plays in the History of Dental Caries." In the few comments made on the latter article, the writer pronounced the theory "a novel and ingenious one," and though "it lacked sustaining evidence," was in this respect on an equal footing with all other theories advanced upon that subject.

The author of the report next brought up the theory advanced—or rather *statement* made—by Dr. Garretson, in regard to the development of the enamel, in his second edition of "A system of Oral Surgery," which the writer said was new, at least to him. The following quotation was made from that work—page 105–6—and the new theory it contains commented upon :

"The formation of dentine completed, the covering of it with enamel begins; or rather this deposit is, to a degree coincident with the dentinal formation. Secreted by the *same pulp* which formed the dentine, some portion of the *same secretion* finds its way into and through the primary sac. As it passes through this sac it is modified, receives new elements, perhaps, which, as it is received into the second space, or the space between the first and second caps, impresses upon it the arrangement of its particles after the hexagonal order of the enamel."

Although the writer of the report did not agree with Dr. G. in regard to his theory of the development of the enamel, he thought the following facts, as far as they had any significance in relation to the subject, might be adduced in favor of the author's views :

"It is admitted generally," the report says, "that the enamel, *after* the tooth has been erupted, becomes more fully developed; receives an additional supply of calcerous salts, and also, that after the enamel has become fully developed, it is the recipient of nutrient matter. This developing material and nutrient substance must come from the dental pulp. If these may be taken from the pulp *after* the enamel organ has disappeared, it would seem *possible* that it may possess the formative power ascribed to it by Dr. G." "But," the report says, "there are histological difficulties in the way which render this view highly improbable."

The writer then averted to a paper written by himself, and read before the Illinois and Iowa State Dental Societies upon the question, "May the Calcific Elements of the Deciduous Teeth be Appropriated in the Formation of any Portion of the Permanent Ones?"

The writer discussed the subject fully, and these are his conclusions, in his own language: "We have seen from the foregoing that the process of nutrition is carried on in an exceedingly prudent and *economical* manner; that the elements taken from the deciduous teeth are not degenerated materials, nor unfit pabulum for other tissues; that the inorganic substance, common salt, may be used over and over again, subserving an important purpose in nutrition, until it is gradually lost with the excrementitious matters ; that portions of the disintegrated muscles, which are composed almost entirely of organic substance, may be converted into pabulum for other tissues; that the same identical phosphates may be used indefinitely by animals whose food is deficient in these essential ingredients ; and finally—which is a significant fact—we have seen that these lime salts of the deciduous teeth are taken into the circulation at a period of life when these elements are the most imperatively demanded in the *growth* of the young animal."

On motion of Dr. Goddard, action on the proposed amendments to the constitution was made the order of business for 3 P. M.

Adjourned to 2 P. M.

AFTERNOON SESSION.

President Dr. Hunt in the chair.

At 3 P. M. the proposed amendments to the constitution were taken up.

Article III, Section 3, was amended to read as follows: The members of this Association shall be of two classes—delegates and permanent members—having equal rights and privileges, except eligibility to office; none but those who, on signing the constitution and paying their dues, declare their intention of becoming permanent members, shall be eligible to office.

Article IX was amended to read: Amendments may be made to the foregoing articles or rules of order at any meeting if there be no dissenting voice

Article VI, Section 1, was amended by inserting in the list of standing committees, the word Aetiology.

Article V, Section I, was amended so that the three names, or in case of a tie, the four names, having the greatest number of votes shall be the sole nominees in case of a failure to elect on the first ballot.

The following resolution passed at the last meeting, was rescinded: Henceforth no person shall be received as a delegate who is in arrears for dues, or until they have paid to the Treasurer the full amount due at the time their names were dropped for non-payment.

Dr. H. Judd, chairman of the Committee on Pathology, made a report which was enthusiastically received. Dr. Atkinson congratulated the Association on the report just read. It evidenced a degree of progress in this branch which was exceedingly gratfying to him; did not agree with the author on some few points, took exceptions to the pronunciation made use of in the report of the word Lucocytes, giving its derivation from the Greek, together with his views of its pronunciation ; gave the successive steps in the formation of organized tissue from the atom, and detailed an experiment showing the power of dessicated corpuscles to reproduce themselves.

The executive Committee reported the following programme :

Hours of holding sessions, from 9 A. M. to 12 M., and from 2 P. M. to 6 P. M. daily, except on Thursday, when there will be no morning session, the time being occupied with clinics ; afternoon session same as on other days, and an evening session from 7.30 to 10 P. M.

Adjourned to 9 A. M.

### Morning Session.

9 A. M., President in the chair.

The committee appointed at the last meeting to prepare a circular on dental education being called, Dr. Judd said he had no report to make ; Dr Dean had not prepared the circular, and did not think Dr. Palmer would be present at this meeting. On motion the time of the committee was extended to the next meeting.

In the discussion of the report on Pathology and Surgery. Dr. Judd said : Dr Atkinson has taken pains to correct my pronunciation of the word Lucocytes ; he seems to think it should be Lucocytes; if it were purely a Greek word this would undoubtedly be correct ; but being an Anglicized Greek word, it should, I conceive, be pronounced Lucocytes, the plural of Lucocyte.

His description of the blood corpuscles under the microscope was good ; his observations, I am inclined to think, were of dead corpuscles. There is some difference between his opinion and my own in regard to dead matter. After certain characteristics which distinguish living from dead matter cease to be, it becomes dead matter. As to the dead dessicated corpuscles being capable of being revived and reproducing themselves, I have my doubts.

Dr. H. S. Chase read a report on Pathology.

Dr. Chase's paper opened with a case of destruction or absorbtion of the alveolar processes, with their subsequent restoration after three months' treatment.

The patient was " aged 19 ; short, stout body, fat, florid and somewhat coarse complexion, black hair, black eyes. About three years ago she was sick with intermittent fever for a year ; does not know whether she took mercury or not."

At the end of that time her gums began to swell, and a discharge of a thin character took place from their under surfaces.

" Present appearance, April 17th : The gums are of a purplish red color, over all the teeth of both jaws ; they are everywhere hypertrophied ; from their under surfaces flows a viscid, purulent fluid, bathing the teeth and smelling offensively. The teeth are all present in the mouth and undecayed, but very loose. With a flat probe I can pass up under the gums towards the ends of the teeth about half an inch before meeting the alveolar processes, and can pass the instrument entirely around the roots of the teeth."

The patient partakes freely of meats, coffee, pepper, spices, pickles, mustard and salt.

Treatment : Diet to be changed ; stimulants forbidden ; plain milk food only, allowed. Local treatment by cauterization with " chloride of zinc paste ;" viz : chloride of zinc crystals, 60 grs., and powdered Blood-root 15 grs., ground without water to a paste.

Nitrate of silver was tried on the left side of the mouth at the same time that the chloride of zinc was used on the right side, and in the same manner ; but after two applications, as the chloride was found to be doing good work, while the nitrate was showing no good results, the latter was abandoned. About ten applications were made in twelve weeks. The paste was carried up on a thin instrument and left in contact with the gums and alveolar process, and roots of the teeth.

There had been no recession at all of the gums This was *not* a case of TARTAR. A hardly appreciable quantity of some salt was found on the roots at every visit, and was as often removed. At the third visit, this incrustation was put under the microscope, after drying, and large numbers of what appeared to be chloride of soda crystals were seen, together with amorphous granules. The case improved after the second visit, and continued to do so until there was a complete restoration of the alveolar processes, and the teeth had become *tight* again.

At the close of his paper Dr. Chase said: "But the Physiologist can draw no line of demarkation between health and disease;" "only when he observes marked difference between them, can he say, 'this belongs to the domain of pathology, and this to physiology,'" "Who can draw the line between the animal and vegetable kingdoms?" Microscopic research shows that living beings are evolved out of the non-living, independently of the influence of living matter;" "the same living matter may grow into both vegetable and animal forms." "Life is an unceasing round of chemical actions, expressing itself in various forms from the highest to the lowest, depending on its surrounding incident forces." Dr. Chase thinks that small-pox, syphilis, scarlet fever, measles, &c., can arise *de novo, now*, as well as they could in the *beginning*."

"The *diseased* living matter which has the power to be transferred from one living organism to another, and there multiply by the plasma of the blood which it feeds upon, is not to be distinguished in form or chemical composition from healthy matter." "May not this power to do mischief be owing to the peculiar arrangement of its organic molecules? There are many inorganic compounds having the same chemical composition, precisely, that yet have entirely different forms and powers. This difference in properties is supposed to depend on the peculiar arrangement or juxtaposition of their ultimate atoms."

Dr. Chase thinks that there will yet be a host of new diseases arise *de novo*, or be evolved out of pre-existing diseases, as the conditions produced by social life, density of of population, &c., vary.

He also says: "The future looks bright with the promise that vital chemical changes, both in health and disease, will be as well recognized as those which take place in the laboratory of the chemist." "The physician will then be able to select and apply remedies for disease with as much precision as the chemist can now select elements to form definite compounds, and again to separate the most complex compounds into their elementary individualities."

Discussion.—Dr. Judd—Of the paper to which we have just listened, in relation to the case of absorbtion, I desire to say a word. I witnessed some of the treatment, which was very interesting to me. I have seen several such cases within the past few years. I took pains to examine microscopically the discharges from beneath the gums in this case. I found that when there was but slight appearance of disease, by passing the probe up underneath the margin of the gum I could get pus corpuscles, the result of inflammatory action there. I have used both of the remedies spoken of, and was glad when this opportunity was presented of demonstrating the efficacy of these agents at the same time and in the same mouth. I had a similar case a few years since, which I treated and discharged, apparently cured. In time it returned, and a renewal of the treatment cured again. A slight constitutional disturbance would reproduce the disease. In relation to the microscopical characteristics of the incrustations found on the teeth, resembling the chloride of lime crystals, or whether it was identical with the deposits commonly found on the teeth, I cannot say. The doctrinal points put forth in the paper are very important. The doctrine of spontaneous generation, the evolution of the living from the non-living, is a matter of much moment. The doctrine is an ancient speculation which proved unanswerable till the seventeenth century. Francisco Redi, an Italian physician, was the first to announce the doctrine that all living matter has sprung from pre-existing living matter. It was universally believed that maggots were generated in dead flesh; but this was disproved, by placing animal body, when quite fresh in a jar, and excluding external agencies by tying fine gauze over the top of the jar, when not a maggot was found, although putrefaction went on as before. The disciples of this old theory have been driven from point to point till they have little left to base their theory upon. I do not believe in the doctrine, neither do I believe it to be the general belief of scientific men at the present day.

Dr. Abbot—This case reported by Dr. Chase is a very interesting one. He does not say that he restored, or thought he restored, the periosteum. When this tissue is lost I do not believe it can be restored. I find teeth loose from hypertrophy of this membrane, surrounding the roots, the result of irritation, produced by the deposit upon the teeth; remove the cause and we cure the disease. It is not necessary to run the chloride of zinc or nitrate of silver up there; nature is her own physician. In my own practice I find nothing necessary but a dressing of carbolic acid. I introduce an instrument under the margin of the gum and remove the dead border of the process, and restoration takes place; making use of a dressing of carbolic acid and water, one drachm of acid to eight ounces of water.

Dr. Walker desired plain, common sense statements, and not vagaries which nobody understood. His treatment in cases of this character was to apply the chloride of zinc diluted with water, equal parts, till the disease was stopped, and then apply carbolic acid.

Dr. Osmond.—I content myself with simply using a wash composed of chlorate potash two drachms, tinct. myrrh three drachms, rose water six ounces.

Dr. Morgan.—We all seem to have our own peculiar mode of treatment in these cases; if this is the case we are the greatest set of empirics the world ever saw. I did not think this could be true, but I now begin to believe it. The periosteum may never be reproduced on the root of a tooth, but I am confident it is in some other localities.

Dr. Herriott.—I am much pleased with the paper. I

think Dr. Abbott is wrong in saying that the periosteum can never be reproduced. I am satisfied that no explanation can be given for the conditions we find resulting from correct treatment of these cases, if we do not recognize the reproduction of this membrane. The systematic treatment adopted in the case reported was the cause of success. We may have different modes of treatment and not be empirics.

Dr. Judd.—I arise to correct certain misapprehensions which seem to exist in the discussion of this case. In this case there was no deposit of tartar, as we find in simple cases of recession of the gums. In this case the immediate cause of the inflammation had passed away.

Dr. Buckingham.—I think the remark of Dr. Morgan, that we are a set of empirics, was correct. What is the effect of chloride of zinc. and what is needed in a given case ? We must know this if we would work understandingly. You must exclude the parasites before nature can heal the wound. If this is so the chloride of zinc is demanded, or any other anti-septic may be used; nature requires it.

Dr. Stellwagen.—I think we are wandering from the subject. Dr. Chase did not go into the details as to how the chloride of zinc acted; he removed the foreign matter and then used the chloride of zinc to produce a contraction of the space around the roots, so as to exclude the foreign matter.

Dr. Abbott.—I do not think I made a mistake in asserting that the periosteum cannot be renewed. I would like to have Dr. Chase tell us if he thinks it was in the case he has reported.

Dr. Chase in reply to questions.—I used the term atoms in referring to inorganic matter, and the term molecules to organic or living matter. The deposit was soda crystals. I carried the instrument up about five eighths of an inch; not above the point of the root. I did not know whether I removed the periosteum or not, and did not care. I knew the teeth would become firm if I removed the disease.

Bastian has proved that it does not need air or oxygen to produce fermentation. It has been asserted here that Pasteur and others had made the disciples of this doctrine of Bastian's hide their heads. We know that Bastian is sustained by many in the scientific world, and that his experiments go far to prove the correctness of his theory. He partially filled a glass tube with a fluid, heated it up to 300° and then sealed it hermetically. In a few days he found Bacteria and other forms of living matter in the fluid. An infusion of turnips was heated in the same manner, and in a few days Amoeba was found; bits of living matter thus produced develop into monads with tails—tails that will wiggle. No matter if Pasteur does not succeed in obtaining these results; Bastian did not get them in his first experiments. Huxley says if he could look back into a far distant geological age he would expect to see microscopical forms growing out of inorganic matter.

Dr. Judd.—The defense made by Dr. Chase has been as good as could well be made. He has put forth the doctrines and arguments of those who believe in this theory. All of these experiments of Bastian's have been made in the supposition that 300° of heat destroys these germs. Pasteur has demonstrated that this is not true.

Adjourned to 2 P. M.

### Afternoon Session.

2 P. M., President in the chair.

Discussion on Pathology and Surgery continued.—Dr. Stellwagen.—Referring to the terms periosteum, peridenteum and pericementum: After inflammation has taken place in the periosteum I defy any one to show me a line of difference between the periosteum and pericementum.

Dr. I. S. Smith, of Pennsylvania, thought root-membrane as good a name as any for this tissue.

Executive Committee report clinics at 8 A. M. to-morrow: Dr. Corydon Palmer, Operative Clinic; Dr. C. R. Butler, Diagnostic and Operative Clinic; Dr. W. Atkinson, Diagnostic Clinics.

Report on Therapeutics by H. L. Sage.

In speaking of the phosphates he said: The French physicians claim that the lacto-phosphate of lime is the form most readily taken up and appropriated by the system. When the gastric juice is concentrated it readily dissolves the phosphates. This remedy would seem to be specially indicated in cases of females during the periods of gestation and lactation, when the stomach is in a feeble and irritable condition. There are two classes of theorists in regard to the use of lime salts, one recommending the use of the phosphate in cases where there is a deficiency in the lime salts, and another would use the lacto-phosphate. The following formula for a syrup of the phosphate was recommended: Acid phosphoric dilute U. S. P. q. s., phosphate lime, fresh precipitate, q. s. Dissolve the phosphate of lime to saturation in the acid, filter, and add an equal quantity of pure glycerine or sugar—the glycerine is preferable. In case of anæmic condition, phosphate of iron may be added with advantage, also made tonic by the addition of sulph. quinia. Register 71.

In cases of retarded dentition it is especially indicated. This should not be confounded with checked development, as there is a great difference between these conditions in cause and result.

Another direction in which the lacto-phosphate seems to be indicated is in cases of irregularity in the dental arch; in many of these cases there seems to be a want of lime salts, and in correcting the irregularity constitutional treatment is indicated. The administration of the lacto-phosphate furnishes lime for filling up the spaces formed by the side of the root changing its position.

A new agent (pepsin) has been suggested for use in the treatment of dead and partially decayed pulps, by Mr. Oakly Coles, of London.

In alluding to the injurious effects of certain remedies on the teeth, he stated that acids which are known to have a very injurious effect on the teeth are frequently administered without proper care. Remedies containing potash, as bromide of potassium, have greatly impaired the teeth when long continued. A case came under my observation where this remedy had been taken for one year. The teeth were found very much denuded. When the saliva is acid the salt is decomposed and bromic acid is formed, which is very destructive to the teeth.

Carvacrol.—In referring to this agent the writer stated that carvene, carvol and carvacrol are products of the essential oil of caraway, which is isomeric with the oil of turpentine. The oil of caraway consists of two liquid oils; one a carbo-hydrogen called carvene, C 20, H-16, and the other composed of carbon, hydrogen and oxygen, C-20, H-14, O-2, and named carvol, the boiling point of each being respectively 343° and 472°, Fahr. When the oil of caraway is distilled from hydrated phosphoric acid, the distillate being poured back into the retort until it ceases to have the smell of caraway, the product is an oily liquid called carvacrol, having a disagreeable odor and a strong, acrid and very persistent taste. (U. S. Dispensatory, 18½ page 1304.) Carvacrol is obtained by treating oil of caraway with potash, or again by treating the same oil with iodine, cohobating several times, and washing the product with potash. Thus obtained it is mixed with carvene. Carvacrol is also found among the products of the action of iodine on camphor. Carvacrol, when pure, is a colorless, viscid oil, lighter than water, in which it is nearly insoluble. The odor of carvacrol is like that of creosote; taste, persistent, strong and biting. It is a mild antiseptic, carminative, sedative and stimulant. Combined with water it forms a very pleasant and efficient gargle for tonsellitis. Also, as a mouth wash, it is very excellent. In cases of odontalgia, from an exposed pulp, it is singularly adapted, its application giving almost instantaneous relief. It is useful for rinsing the nerve canals and as a dressing in cases of alveolar abscess. It does not produce as much inflammation in the surrounding tissues, when applied through the canal, as creosote or carbolic acid, and yet it makes itself more severely felt. Oxychloride of zinc is the best material for a temporary covering of this substance, as Hill's stopping, or gutta percha, is speedily dissolved by it.

Discussion.—Dr. Atkinson.—The assertion that carvacrol is better than thymol as a disinfectant, or as an agent for obtunding sensibility is unwarranted. The shock produced by the glycerole of thymol is due to the glycerine. There is no shock where there is no excitation. Pure thymol produces no pain. If we forego the pain attending the application of the glycerole of thymol we shall have an agent that meets the case as a disinfectant and obtundent of sensibility. I first used creosote, then thymol, and now carvacrol. As a substitute for creosote, carbolic acid and thymol I am not prepared to accept it. They are all good and similar in character, the carvacrol being the mildest in its action. In reply to a question: Pepsin will not dissolve anything; under a certain kind of stress it stands there and enables water to dissolve the albumen. I endorse Mr. Cole's method of using it. Apply it and it will wash away the decomposed nerve.

Dr. Taft uses the pure dried pepsin, forming a paste with the liquid preparation. Has used carvacrol and finds it good—thinks it promises much. The subject of therapeutics is far less understood and is receiving less attention at our hands than the operative and surgical branches. This is to be regretted. There is very much that pertains to this branch of medicine that is of vital importance to the dentist, and should be better understood. An attempt has been made to simplify the knowledge necessary for the proper administration of most of the principal remedies by putting them up in convenient form, ready for use, classified and so marked that the amount given is always indicated. Opium, for instance, is combined with gelatin in thin sheets, and so marked that a large or small portion can be used as desired. All non-evaporating medicines are prepared in this way.

On motion of Dr. Rehwinkle, it was resolved that the

Committee on Therapeutics investigate the difference of action between creosote, carbolic acid, thymol and carvacrol, and indicate symptoms or peculiar conditions under which one of these agents is preferable to the others.

Dr. Stellwagen uses alcohol perfumed with cologne as a disinfectant and dressing for root canals. Thinks it is not necessary to use any thing else—too much stress is put on this question of proper remedies for the treatment of diseased dental tissues. In reply to a question: He never uses chloride of zinc in filling a root canal at the apex; sometimes uses cotton saturated with carbolic acid, sometimes gutta percha, and sometimes gold.

The subject of Therapeutics—passed.

Dr. J. B. Hitchcock, Chairman of the Committee on Histology and Microscopy, made his report.

Commencing with the development of the teeth, the writer gave the successive steps in the process of the development of the teeth, from the primitive dental groove to the fully developed organ. As this portion, as well as the concluding portion of this report, which was devoted to the histological structure and microscopical appearance of calcific deposits found in the pulp canals of human teeth and the teeth of animals, was mainly descriptive of a series of photo-microscopical plates which the author had prepared, we are not able to make any extended report of it. We hope arrangements will be made for supplying the publishing committee with copies of the plates, to accompany the report in the published transactions.

Dr. Taft introduced Dr. Eastlake, resident dentist from China.

Dr. Judd.—In relation to these calcific deposits and new formations mentioned in the report on histology: These formations are sometimes continuous with the dentinal structure, at others connected only with the pulp chamber or nerve canals. There is a characteristic difference in the structure of these formations, apparently dependent somewhat upon their locality. Those found in the pulp chamber approach more nearly to the tooth structure than those connected with the dentine. I have seen specimens containing well developed dental tubes; others seem to possess no characteristics of the dentinal structure. A recent specimen which I prepared and examined, had all the appearance of dentinal tubes arranged in regular order, and as distinct as I have seen them many times in a healthy tooth. The influence these deposits have upon the dental pulp is various, many times causing intense neuralgic pains. These nodules in the pulp chamber are found quite frequently in perfectly sound teeth. In reply to Dr. Taft: I would open the nerve chamber and remove the nodules, treat as in simple exposure, or remove the nerve entirely and fill. Sometimes these formations extend up into the canals, making it very difficult to remove them.

Dr. Morgan.—As Dr. Judd has said, these nodules are frequently found in the nerve canals, and I think I have noticed that the pain consequent upon their presence is more acute when the nodule is connected with the dentine than when connected only with the nerve.

Dr. Atkinson.—I desire to congratulate this body upon the direction that thought and investigation is now taking. There seems to be a manifest desire to seek out and know the source and cause of things in nature.

The hypertrophy which has been spoken of as being present in the cementum of a tooth, is due to the want of occlusion of this tooth with its fellow.

Dr. Chase has specimens of dentinal tubes extending half way up to the surface of the enamel, and several specimens of calcified pulps; also a specimen of decalcified crown of a tooth in which there were osteal cells at the end of dentinal tubes.

### Afternoon Session.

2 P. M. In the absence of the President, Dr. Morgan was called to preside.

The following resolution, offered by Dr. Walker, of New Orleans, was adopted:

*Resolved*, by the American Dental Association, convened at Put-in-Bay, that the recent action of the railroad combination, in refusing to make any reduction in fare, as far as it applies to dental, medical and scientific associations, is not only uncalled for but highly reprehensible.

*Resolved*, That gentlemen who attend such associations as delegates, do so at great personal sacrifice for the benefit of humanity and the general public good, and that their influence tends greatly to advance the interests as well as the area of civilization, and thus confer such positive benefit upon railroads (which depend upon the increase of civilization for their welfare,) that they can well afford to pass such members and delegates both to and from their places of meeting, and that their refusal to grant at least return passes, is laying a tax which they are illy able to bear, upon men who devote much time and labor without compensation for the public good, and is as short sighted as it is arrogant and selfish.

Dr. Atkinson read a volunteer essay on Histology. This was an able paper, and we regret that we have no report of it. After the reading of this paper the subject of Histology was passed.

By request of the association, Dr. Eastlake gave a brief history of the status of dentistry in China and Japan. The speaker said: You cannot expect me to give you a very favorable report of the status of the profession in this hot-bed of superstition, or that I can give you anything new, isolated as I have been for twenty years. I arrived in Hong Kong in 1853. I found dentistry at a very low ebb. The inhabitants, a superstitious people, educated to believe in charms and necromancy, did not appreciate dentistry. Since I first landed there has been a great change in the character of the inhabitants--brought about mainly by the English with whom they have had intercourse. It would be difficult to find a country where dentistry is more appreciated than it is in Hong Kong at the present day. I have had as patients, representatives of all the Eastern nations. I have had the same difficulties to overcome that you have in the treatment of diseased teeth. The Chinese dentists are a set of charlatans, jugglers and mountebanks, who work upon the credulity of the people, extract teeth without pain, and perform instantaneous cures through the aid of necromancy. I was dependent upon my own resources—made my own instruments and outfit. To give an idea of the Chinese dentist, I will relate an incident: Some of my patients desired to know how it was that the native dentists were able to extract teeth without pain, and how they removed the worm from the tooth which they were taught to believe was the source of pain. That I might be able to answer some of these questions, I visited a dentist. I found him in his tent, or under his umbrella, in front of the temple of horrors. Around him were a throng of natives, afflicted with all manner of diseases. The dentist received me kindly, exhibited his instruments, and showed me his method of operating. By close observation I learned many of his tricks. When a patient presented himself to have a tooth extracted, the dentist placed around the tooth upon the gum, a white powder, which he said was the extract of the blood of the horse. The patient then left to return again in a few days to have the tooth removed. When he returns, the tooth being loose, is readily removed without force or pain. The powder used was arsenic. To learn the maggot or worm trick, I was compelled to take one of his instruments used for removing the worm. It resembled a pair of forceps. I found one side of the instrument to be of bamboo, which was hollow; in the hollow were secreted the maggots. The dentist would examine the aching tooth and pronounce it a case of worm in the tooth. Taking his magic instrument he would apply it to the tooth, at the same time forcing one of the maggots out of the bamboo side into the cavity. The patient would then be exhibited to prove the correctness of his diagnosis. Another application of the instrument and the worm was removed and the patient sent away cured. Those engaged in the manufacture of artificial teeth are very dexterous in carving. Their shops are located along the streets, where they are at all times prepared to insert one or more teeth at short notice and for a small fee. The patient is placed on a table and a piece of ivory or hippopotamus tusk is selected and carved to fit the space, and to resemble a tooth; it is then attached to the natural teeth by means of copper wire. I have seen specimens of this work which had been worn for years. A man in Yeddo carves plates for sets of teeth from a hard wood, resembling mahogony; human teeth are then attached to these plates by means of rivets. Colleges and schools are to be found all over Japan. The teeth of the Japanese are of a superior character—much finer than our own. The better class are very cleanly—to keep their teeth clean is part of their religious duty. They perform this duty every morning and night, before going to prayers. They first clean their tongue, then their teeth, and then say their prayers. To clean the teeth they use a piece of wood with a powder made from the cuttle fish. Caries is not common except among the coolies.

The following resolutions, offered by Dr. Rehwinkle, were adopted:

*Resolved*, That this Association, recognizing and appreciating the liberality of the Cincinnati, Hamilton & Dayton, Michigan Southern, Cleveland, Columbus, Cincinnati and Indianapolis, and the Short Line railroad from Louisville to Cincinnati, in granting round trip tickets to and from Put-in-Bay to members and delegates of the American Dental Association, hereby expresses its sense of obligation to the above named roads.

*Resolved*, That in view of the fact that an urgent necessity does exist to supply the army and navy of the United States with competent dental surgeons, it is hereby proposed that a committee of three be appointed to secure the co-operation of the Surgeon-General of the United States army, and if needful of the Congress of the United States, to make provisions for the appointment of dental surgeons in both army and navy.

Dental Chemistry.—Dr. Webb read a report on this branch, mainly devoted to the physiological action of anæsthetics. It was a good paper, and we regret that we can not give our readers a synopsis of the writer's views.

Dr. Atkinson, referring to the paper, said: The deprivation of oxygen is the cause of anæsthesia. Functional action does not take place in statical tissue. I wish some one would speak on the subject of chemistry directly, we should then have something on crystalography; something upon which we might learn a thing or two of calcification and absorbtion of the teeth.

Dr. Buckingham.—That all anæsthesia is the result of the want of oxygen is an old theory. If it were true, then we might give hydrogen, nitrogen, or any other gas which has no oxygen. I think anæsthetics act by being passed through the blood and being absorbed, and so act upon some of the functions of the body as to deprive them of the power to act. I know not how.

Dr. Webb was asked by Dr. Atkinson if he understood him to say that the blood corpuscles have a sac; if so, he would advise him to investigate the matter for himself, and not take the say-so of others. He doubted it.

Dr. Atkinson was asked if his theory be the correct one, how he would account for the anæsthetic effect of mesmerism. In reply Dr. A. said, "mesmerism affords the best evidence that can be adduced in support of this theory. In the mesmeric act the mind must be kept fixed and at rest; respiration and inspiration are gradually arrested, and a general cessation of feeling and sense ensues. Awaken the mind, restore the function of respiration and inspiration, and general restoration soon takes place. Mrs. A— had the power for three days, and everything that could be done was done by physicians to awaken her, but without avail. A brother in the church suggested that they give her a song of Zion; it was tried, and sister A— was alive in no time."

Dr. Summers.—I do not believe it is the deprivation of oxygen that produces anæsthesia. In fact, I know it is not.

If you take oxygen from the system you do not produce anæsthesia. Alcohol has been suggested here as an agent for cleansing root canals We have no better disinfecting agent than pure alcohol. I use it in the laboratory for this purpose. For preserving meat from putrefaction and maggots it is the best thing I have found. With reference to the action of acids and alkalies upon the teeth in the mouth, I am still of the opinion that the alkalies are the most destructive agents. They act upon the pabulum, taking away the base. The presence of acids does not prove that they are the cause of decay. There is nothing so destructive of animal life as the alkalies. Hydrochloric acid acts slowly upon the teeth, but we do not carry enough of this acid in the mouth to destroy the teeth.

Dr. Buckingham.—Alkalies may destroy the teeth by overcoming vital force and by causing the elimination of an acid destructive to the teeth. Alkalies will not destroy a tooth if immersed in them.

Dr. Summers.—These alkalies enter the blood vessels, thus coming directly in contact with the tooth substance through the local circulation, and so destroy the pabulum of which the tooth is formed.

Dr. Taft.—Why does it not act upon other bones and tissues, if it goes into the blood?

Dr. Summers.—It goes into the blood at a point immediately surrounding the teeth, through the corpuscles, and destroys the pabulum.

Dr. Taft.—I apprehend that any agent passing into the general circulation would affect the general system. The teeth do not get their supply of nourishment from the corpuscles immediately surrounding them.

Dr. Atkinson.—I think the truth will be found in the mean between the two theories. Alkalies enter the tooth at the distil end of the tubuli and interglobular spaces, and act upon the tissue. Its affinity for the pabulum causes it to attack the teeth in passing the interglobular spaces, being attracted by the pond of supply.

Dr. Osmond.—I have observed the evil effects of alkalies on the teeth. By the constant use of baking powders and soda the phosphates are eliminated and the pabulum destroyed. In tuberculosis the phosphate of soda has been found to be of great service. The rationale of this treatment being to supply the waste of the phosphates. Baking powders rob the system of the phosphates and render the secretions acid, thus destroying the teeth. Sozodont should not be used. If we wash our hands with soap repeatedly and thus remove the cuticle, it will be reproduced; but if we remove the corresponding tissue from the teeth, it cannot be restored.

Dr. Douglass.—I prescribed iodide of potash for a patient; after taking it some time the teeth became very sensitive, and the gums commenced receding. The use of the remedy was discontinued and the local trouble disappeared. The same patient took bromide of potash recently, and the difficulty with her teeth and gums returned.

Dr. Osmond.—I would recommend the application of nitrate of silver for this sensitive condition of the teeth; two or three applications will cure the disease. It will blacken the teeth but it renders them harder. I have good success with this remedy. To apply it I make a small disk of parafine, and drop a drop of the nitrate of silver into it. I use a common tooth-pick to apply it with.

Dr. Buckingham.—A wire of silver placed in nitric acid and applied at once, gives you the nitrate of silver in as convenient a form as possible.

Dr. Hitchcock.—Teeth with perfect enamel are not affected by the alkalies. If decayed or denuded of enamel, the alkalies may render them sensitive. I have a tooth which has been immersed in a solution of soap for two years, without the least change—at least in the enamel. I have used soap in my own mouth for years, with no unpleasant effects. I recommend it for my patients. I have used the nitrate of silver when there were symptoms of decay, teeth sensitive, not much softening, applying it only to the defective spot. I use the solution, and apply it with a stiek, following it with a solution of the carbonate of soda, to destroy the metallic taste. Chloride of zinc has been used in similar cases, and is good. The teeth of females are frequently sensitive during the period of gestation, especially about the necks. If we apply litmus paper, we shall find the secretions at this point acid. Excavating gives pain. The bicarbonate of soda, one half teaspoonful, given three times a a day will remove the trouble. The sensitiveness will disappear and the teeth can be excavated with little pain. I would only fill the teeth temporarily during these periods.

Dr. Hunter.—Alkali, if I understand Dr. Summers, destroys the framework of the teeth, and allows them to break down. This is contrary to the recognized theory that the cause of decay is external and not internal. I have never seen a case where I thought the cause was internal.

Dr. Rehwinkle.—If Dr. Summers' theory be correct it is certainly very important, and a great boon; for if the capillary system possesses the power of absorbing any agent, and of carrying and applying it to any specific organ, we have a powerful adjunct in overcoming disease.

Dr. Summers.—I hope no one thinks I am so ignorant as not to understand that any substance introduced into the circulation traverses the entire circulating system. Still, might not a substance be introduced into the capillaries around the tooth, so affect the blood that goes to nourish the tooth as to affect the tooth structure?

Dr. Buckingham.—But the tooth does not get its nourishment from the external vessels surrounding it, but from the blood. I use alkalies to destroy the sensitiveness of teeth; I have used caustic potash.

Dr. Stellwagen.—I have tested case after case, and have never found one where the secretions showed an alkaline reaction. I understood Dr. Summers. I fully understand the osmotic action alluded to. There is this action going on, but I do not believe the alkalies produce any effect in this way upon the teeth. I have said that we could preserve our patients' teeth if we could keep them clean, and the saliva alkaline. I announced this at Nashville, and believe it still. Use chalk around the teeth at night, and give lime-water and milk to correct any tendency to acidity. I have adopted this course in my own family, and when we neglect it for a little time decay runs riot.

Dr. Whitney, Honolulu—Burials in the Sandwich Islands were formerly made in caves. These burial places are accessible, and in them may be found the skeletons of former generations. At the time these burials were made no acid fruits were to be found in the islands.

They have since been introduced, and the date of their introduction being known, it occurred to me to make some examination of the skulls and teeth of those who died before and after the introduction of the acid fruits, and institute a comparison between them, with a view of ascertaining, if possible, the effect, if any, that the use of acid fruits have upon the teeth. I found on examination that decay was present before the introduction of acid fruits, but not so marked as it is now, and has been since their introduction. I find a gradual increase of decay with the increased use of acid fruits.

Dr. Forbes.—There is nothing more troublesome and annoying to the dentist than sensitive dentine. There are very few of us who can say to our patients as Dr. Arthur says he does: "If you do not eat and drink as I direct I will not treat you." We have had alkalies, caustics and acids recommended to-day, for obtunding sensibility. I have used alcohol with common chalk, and I find in its use all that has been recommended, for it is well known that manufacturers put nitric acid in their whiskey. This is my tooth powder: prepared chalk and alcohol. The more simple the injunctions the more likely they are to be carried out. My patients get married and their teeth are improved.

Dr. Druylass.—I do not believe that alkalies, in small doses, will produce recession of the gums, or decay of the teeth; but given in large doses, or for a long time, they may produce these conditions. Sour drops will produce peridental gingivitis. When produced by alkalies, use nitric acid in high dilution; when from acids, use potash in the same manner. Subject passed.

Dr. Hitchcock opened the subject of operative dentistry with the details of several cases of torsion. He said: There is an operation which has been performed in Europe frequently, which is worthy of our attention. I refer to torsion as a means of correcting the irregularities of the dental arch. The method of performing this operation is pretty fully described in Tomes' Dental Surgery—second edition. It should never be attempted during certain febrile conditions. There should be plenty of space to turn the tooth without impinging upon others. It should be done soon after eruption, before the root is fully formed; if not done then there is danger of constricting the vessels. Between the ages of 9 and 12 years is the most favorable age, although it has been done at a later period. Our English brothers do much of this work. If much change is required in any given case, but one half is done at the first sitting; in a few days the patient returns and the operation is completed. The Dr. exhibited models of several cases in which he had performed this operation; all successful save one, a hospital case, in which there was no plate used to retain the tooth in position. A pair of straight incisor forceps with sharp edges, is the instrument used in this operation. Bits of crocus of sand paper are placed between the beaks to protect the tooth; chamois skin or tea lead will answer the same purpose.

Adjourned.

8 P. M., Thursday. Dr. Morgan in the chair.

The first order of business, the selection of a place for holding the next annual meeting, was taken up. Several places were named as suitable, and were balloted for. Detroit receiving the greatest number of votes, was announced as the place selected for holding the annual meeting in 1874.

The election for officers to serve the ensuing year resulted as follows:

*President.*—Dr. T. L. Buckingham.

*First Vice-President* —Dr. Isaiah Forbes.

*Second Vice President.*—Dr. A. F. McLain.

*Corresponding Secretary.*—Dr. Jas. Taft.

*Recording Secretary.*—Dr. M. S. Dean.

*Treasurer.*—Dr. W. H. Goddard.

*Executive Committee.*—Drs. Field, Thomas and Cushing.

*Publishing Committee.*—Drs. H. A. Smith and George H. Cushing.

STANDING COMMITTEES.

*Physiology.*—M. S. Dean, E. A. Bogue, A. W. Harlan.

*Pathology.*—H. Judd, H. S. Chase, C. C. Knowles.

*Histology and Microscopy.*—J. B. Hitchcock, C. E. Latimer, J. Taft.

*Chemistry.*—H. A. Smith, C. R. Butler, W. T. Wallace.

*Therapeutics.*—T. C. Stellwagen, L. Jack, J. McManus.

*Operative Dentistry.*—L. D. Shepard, G. C. Dabott, G. L. Field.

*Mechanical Dentistry.*—S. B. Brown, J. F. Canine, E. D. Swain.

*Education.*—A. F. McLain, E. J. Waye.

*Literature.*—W. H. Eames, Chas. Baffett, P. G. C. Hunt.

*Etiology.*—J. H. McQuillen, M. H. Webb.

*Prize Essays.*—Isaiah Forbes, W. H. Goddard, G. F. S. Wright.—*Missouri Dental Journal.*

---

